# Thirdhand Smoke Knowledge, Beliefs and Behaviors among Parents and Families: A Systematic Review

**DOI:** 10.3390/healthcare11172403

**Published:** 2023-08-27

**Authors:** Valentina Vanzi, Flavio Marti, Maria Sofia Cattaruzza

**Affiliations:** 1 IRCCS Bambino Gesù Children’s Hospital, 00165 Rome, Italy; 2 Department of Health Profession, San Camillo Forlanini Hospital, 00152 Rome, Italy; flavio.marti@uniroma1.it; 3 School of Nursing and Midwifery, Faculty of Medicine and Psychology, Sapienza University of Rome, 00189 Rome, Italy; 4 Department of Public Health and Infectious Diseases, Sapienza University of Rome, 00185 Rome, Italy; mariasofia.cattaruzza@uniroma1.it; 5 Italian Society of Tobaccology (SITAB), 00136 Rome, Italy

**Keywords:** thirdhand smoke, parental belief, behaviors, knowledge, child health

## Abstract

Families play a primary role in protecting children. Thirdhand smoke (THS) is an underestimated public health issue, and health effects correlated to its exposition are emerging. The aim of this systematic review was to analyze papers focusing on knowledge, beliefs, and behaviors regarding THS among parents, families, and caregivers, published until May 2022 on MEDLINE, CINAHL, EMBASE, and the Cochrane Library. Critical appraisal of the included studies was conducted by two independent reviewers using the Joanna Briggs Institute Critical Appraisal tool. Out of the 97 articles, 8 were included in this review (two from the USA, one from Europe, and five from Asia). Although there were no restrictions on the date of publication, all the articles were published in the last 10 years, underlining that THS is an emerging concept. They were characterized by relevant heterogeneity in the study design and assessment tools. The results showed that percentages of parents who believed that THS is harmful ranged from 42.4% to 91%, but parental awareness was not always associated with the adoption of a home- and car-smoking ban or healthy behaviors. Further research is needed to understand the resistance and problems faced by parents who are aware of THS risks but unable to improve good practices.

## 1. Introduction

Nowadays, the effects and negative consequences of tobacco smoke on the health of both smokers and those in their vicinity, especially children and infants, are well known and documented [1,2].

Research priorities have shifted to analyze secondhand (SHS) and thirdhand smoking (THS) [3]. The concept of THS is a relatively new topic in the environmental and public health field [4]. THS consists of the contamination that persists after SHS has been emitted into the air [3]. Specifically, it refers to the tobacco-related gases and particles that are absorbed onto surfaces such as carpets, furniture, walls, blankets, and toys, adhere to indoor dust, or are present on smokers themselves (clothes, hair, and hands) [5,6]. The presence of THS in dust, air, and on surfaces leads to multiple pathways of exposure [4]. Tobacco-related chemical substances can persist for a long period of time (for minutes to months) and can be released back into the air, undergo chemical transformations, and/or accumulate long after a smoking event [7,8]. The accumulation of residual nicotine on surfaces becomes progressively more toxic, reacting with ambient nitrous oxide, ozone, and formaldehyde to create carcinogenic tobacco-specific nitrosamines [9,10,11]. The effects of THS on human health have not been completely studied, but clinical research and biological evidence suggest that THS negatively affects health status. Further research is needed to explain in detail THS exposure’s harmful mechanisms. However, laboratory evidence suggests that THS exposure negatively affects organs and systems in developing organisms. Moreover, in utero and early-life developmental periods represent vulnerable windows of susceptibility for these effects [12]. Ali and colleagues (2021) observed the thrombogenic effect of THS in exposed adult mice, and recently, they documented the prothrombotic phenotype linked to in utero THS exposure, which drives platelets into a state of hyperactivity. This condition leads to enhanced functional responses, like aggregation, in exposed pregnant mice [13,14]. Analogously, Snijders and colleagues (2021) studied the effects of THS exposure in utero and during early life in a transgenic Cdkn2a knockout mouse model that is vulnerable to the development of leukemia/lymphoma. They observed that in utero and early-life THS exposure determined relevant changes in plasma cytokine concentrations and in immune cell populations and increased the leukemia/lymphoma-free survival in bone marrow transplantation recipient mice, potentially caused by THS-induced B-cell toxicity [12]. Martins-Green and her colleagues conducted a study in 2014 to explore the impacts of THS on the liver, lungs, skin healing, and behavior. They utilized an animal model that was exposed to THS in conditions mimicking human exposure. Their findings revealed that mice exposed to THS exhibited changes in multiple organ systems and released levels of NNAL (a biomarker for a specific carcinogen in tobacco) comparable to those found in children exposed to SHS and, consequently, THS. This research established a foundation for further investigation into the harmful effects of THS on humans and emphasized the need for potential regulations to prevent inadvertent exposure to THS [8]. Considering that children, toddlers, and infants have immature respiratory and immune systems, they are more vulnerable and sensitive to the unhealthy effects of THS exposure in places where smoking is allowed, especially in homes [15,16]. Parental smoking represents the primary exposure for children [17]. THS exposure happens through different pathways: dust ingestion, dermal absorption, and inhalation of volatile components [5,11]. Children are a group at high risk in terms of THS exposure because they have a higher respiratory rate and thinner skin [18]. Moreover, they spend most of their time at home, crawling, frequently touching surfaces, having hand-to-mouth behavior and interacting closely with the environment [4,19]. Mahabee-Gittens and colleagues (2019) demonstrated that children whose parents do not necessarily smoke indoors have high hand nicotine levels. This finding outlines how smoking bans do not safeguard against THS exposure, and more awareness of this potentially harmful condition is needed [20]. Recently, Mahabee-Gittens and colleagues (2021) confirmed their previous results through another study aimed to quantify hand nicotine and urinary cotinine levels in children of tobacco smokers. Researchers wiped the palm and volar side of children’s dominant hands and collected urine samples from compliant children. All children had detectable hand nicotine and cotinine. The authors concluded that hand nicotine may represent a marker of overall tobacco smoke exposure in children’s environments, and THS pollution should always be assessed to quantify smoke exposure [20,21]. According to previous evidence and recognizing the primary role of caregivers in ensuring a safe environment for children and infants, a review of the literature was undertaken to synthetize knowledge, beliefs, and behaviors of parents and families about THS’s impact on children’s health. The purpose of this review was to identify papers focusing on knowledge, beliefs, and behaviors regarding THS among parents and families.

## 2. Materials and Methods

A systematic review of the literature was designed. A study was eligible for review if it was published in a peer-reviewed journal and focused on parents’ and families’ knowledge, beliefs, and behaviors about the harmful properties of THS. This review included only those studies that specifically consider the point of view of parents, mothers and/or fathers, grandparents, or caregivers (i.e., it excluded studies considering the general population and health care professionals) and in which there was a clear differentiation between SHS and THS. This systematic review considered both experimental and quasi-experimental study designs for inclusion, including randomized controlled trials, non-randomized controlled trials, before and after studies, and interrupted time-series studies. Analytical observational studies (prospective and retrospective cohort studies, case–control studies, and analytical cross-sectional studies) and descriptive observational study designs (case series, individual case reports, and descriptive cross-sectional studies) were considered for inclusion as well. A 3-step search strategy was used. Initially, a broad search was conducted in both CINAHL and MEDLINE to analyze the text words contained in the title and abstract and the index terms used to describe articles. A second search based on all identified keywords and index terms was undertaken across all included databases ((third hand smoke OR thirdhand smoke OR third-hand smoke) AND (parent* OR mother* OR father* OR famil*) AND (belief* OR behaviour* OR knowledge)). Reference lists of all included articles were scanned to look for literature that had not been obtained previously. The search had no restrictions on the date of publication, type of study, or language. Search engines used included MEDLINE, CINAHL, EMBASE, and Cochrane Library. The literature search was conducted in May 2022. Critical appraisal of the included studies was conducted by two independent reviewers using the JBI approach and JBI standardized tools [22,23]. Any disagreements that arose between the reviewers were solved by consensus or by the decision of a third reviewer. The decisions about the scoring system and the cut-off for inclusion of a study in the review were made in advance and agreed upon by all participating reviewers before critical appraisal commenced. A study was considered eligible for this review if it met at least half of the criteria of the JBI checklist: the cut-off score for the inclusion was set equal to or greater than 4 out of 8 for cross-sectional studies and 6 out of 13 for randomized controlled trials. The PRISMA flow chart 2020 was used to illustrate the flow of information through the different steps and for the reporting of this systematic review [24].

## 3. Results

**Figure 1:**
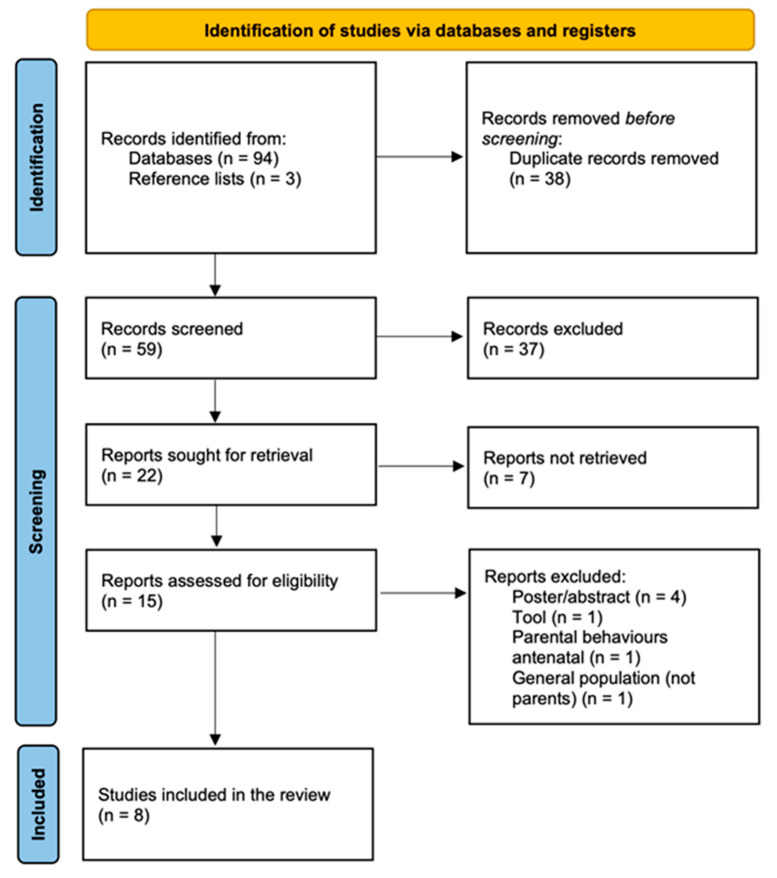
The PRISMA Flow chart.

Ninety-four articles were identified during the initial search. Three additional papers were found by reviewing reference lists. After eliminating duplicates and reviewing titles and abstracts, 22 articles were kept. Based on the full texts, seven additional articles were eliminated. According to the inclusion criteria, eight studies were identified for this review. The PRISMA flow chart of study inclusion is shown in Figure 1.

Overall, there is a heterogeneity of geographical area of publication: USA [17,25], Europe [26], and Asia [18,27,28,29,30]. All papers analyzed the point of view of parents (mothers and fathers), and only one study considered that of grandparents as well [18]. The main characteristics of all the included studies are summarized in Table 1.

In 2012, Patel and colleagues initiated a study aiming to assess the influence of a short THS intervention on the smoking habits of caregivers accompanying children who were admitted to an urban pediatric Emergency Department. The researchers recruited a convenience sample of 40 caregivers who smoke. These parents of children <36 months of age were divided randomly into two groups. One group served as the control and received standard educational information, while the other group received a short educational session about THS. Then, a 6-month follow-up phone assessment was completed at 2, 4, 6, and 24 weeks to register changes in smoking status or policies. Even if the primary outcome of the study was the evaluation of changes in parental smoking status, the paper was included in this review because it analyzed caregivers’ beliefs on THS at the baseline. This study revealed that a quarter of the caregivers disagreed, strongly disagreed, or did not know if THS harmed their children, and half of the responders (n = 21; 52.5%) did not allow smoking in their homes or cars.

In 2014, Drehmer and colleagues gathered information from a nationwide randomized controlled trial known as the Clinical Effort Against Secondhand Smoke Exposure in the USA. They included 1947 volunteer parents as participants in this study, with the aim of assessing their beliefs regarding THS. These beliefs were evaluated through an exit survey conducted after a visit to a pediatric office, involving 10 practices that received intervention and 10 practices that were designated as the control. After 12 months, 1355 parents were contacted to collect follow-up data. The standardized protocol consisted of telephone calls and text messages 12 months after the office visit to ask parents to complete a computer-aided telephone interview. THS beliefs were evaluated at the post-visit enrollment survey and 12 months after the office visit. Almost all responders (n = 1770; 91%) stated that THS is harmful at the baseline, and only a few parents (n = 117; 9%) disagreed. The authors highlighted an association between the belief in the harmful effects of THS and the strict implementation of a smoke-free environment at home and in vehicles, as well as efforts to cease smoking.

Díez-Izquierdo and colleagues (2018) [26] conducted the first European study on parental beliefs about THS, and they enrolled 1406 parents of children under 3 years old in Spain. Almost all of the sample (n = 1209; 86%) believed that THS is harmful to their children, and the authors also identified statistically significant differences in the perception of THS’s detrimental impact based on educational attainment, with those parents who had a university degree displaying a higher level of awareness in this regard.

Baheiraei and colleagues (2018) [27] performed a population-based cross-sectional survey on 1112 families with infants in Iran. They administered a questionnaire through a face-to-face interview with families. Two questions focused on parental beliefs about the impact of SHS and THS on infant health. The results showed that most parents (64.6%) were insufficiently aware of the health consequences of SHS and THS on their children. Additionally, only 42.4% of parents fully concurred with the notion that exposing their children to THS can have harmful consequences on their health.

Moreover, the authors pointed out that a complete smoking ban is not established in many households with infants in Iran.

Myers Gamliel and colleagues (2020) [29] conducted a study with the objective of altering parental viewpoints regarding exposure to SHS and THS. This was achieved by furnishing insights into both exposures, along with individualized data regarding the exposure status of their children. The study involved the participation of 159 parents with children aged up to 8 years. These parents were recruited from Naamat daycare centers, parent groups on social media platforms, and through snowball sampling techniques. Then, they were randomized into three groups: intervention (69), control (70), and enhanced control (20). The enhanced control group was set up to assess the sole impact of measurement without any intervention. Individuals in this group underwent two visits at their homes: during the initial visit, they were requested to fill out the Parental Perceptions of Exposure (PPE) questionnaire, and during the second visit, they were provided with the results of their children’s hair nicotine levels, two informational handouts on tobacco smoke exposure, and answered the final PPE questionnaire. The intervention lasted six months, and it was based on motivational interviews, feedback on home air quality and children’s hair nicotine levels, and educational brochures. Parental perceptions of exposure (PPE) were evaluated through a 23-item online questionnaire, which was composed of eight photographs and nine text vignettes. Adults had to observe each image linked to SHS and THS practices and indicate the level of their children’s tobacco exposition on a scale of 1 to 7 for each item. The score of 6 further questions related to perceived knowledge were added. At the conclusion of the research, the PPE scores had risen in both the intervention and enhanced control cohorts [29]. Two recent studies [18,30] asked parents to complete a web- and paper-based survey, which included the Beliefs About Thirdhand Smoke (BATHS) scale [31]. The BATHS tool is a nine-item scale with a 5-point Likert-type scoring (1—strongly disagree; 2—disagree; 3—undecided; 4—agree; and 5—strongly agree). Tests of its psychometric properties showed that it represents a valid and reliable tool to assess THS beliefs [31]. Shehab and Ziyab (2021) [30] recruited 536 parents in Kuwait with at least one child aged <18 years, and almost all of the sample (n = 459; 85.6%) reported a strict or partial home smoking ban. The prevalence of a strict home smoking ban was higher among never smokers than among ever smokers (49.1% vs. 25.2%, *p* < 0.001). This study showed a good level of parental awareness about the harmful effects of THS on children and adults. Xie et al. (2021) [18] conducted a cross-sectional survey among parents and grandparents of children aged 6–13 years at Changjiang Road Primary School in Shangai. The authors enrolled 843 participants who were asked to fill in the BATHS scale. This study revealed that participants who were aged >65 years were more likely to obtain lower scores on the BATHS scale. Respondents who received higher education were more likely to believe that THS would persist in the environment and impact children’s health. Participants whose children suffered from respiratory diseases in the past six months had higher scores than those whose children had no respiratory diseases. All papers analyzed the point of view of parents (mothers and fathers) and only one study considered that of grandparents as well [18].

Dai et al. (2021) [28] enrolled 145 smoking parents from the outpatient and inpatient pediatric units of the Prince of Wales Hospital (PWH) in Hong Kong by the convenience sampling method. The authors asked them to fill in the KAP scale, a 60-item questionnaire that had a range of 0–38 for knowledge, 0–44 for attitude, and 0–40 for practice. The results showed that most of the respondents (79.3%) had relatively favorable practices regarding children’s SHS exposure, while their practice for THS was inadequate. Parents generally demonstrated better knowledge of SHS than of THS. In addition, the authors underlined the discrepancy between a good level of parental knowledge about the concepts of SHS and THS and an insufficient level of good practice.

The assessment of the methodological quality (or risk of bias) of the included studies was performed using a study design appropriate JBI checklist for analytical cross-sectional studies and randomized controlled trials [22]. Overall, the quality of evidence of the included studies was moderate due to potential biases such as selection, performance, attrition, detection, and reporting biases in experimental studies, as well as due to the risk of selection, information, and confounding biases for observational studies.

## 4. Discussion

THS is an underestimated public health issue, and the potential effects are rising [4]. This review had no restrictions on the date of publication; however, all the articles were published in the last 10 years. This temporal distribution of publications underlines that THS is an emerging concept and a new topic faced in the literature.

Most of the studies were conducted in the Asian Region. This aspect may reflect an increase in research interest and public awareness of the negative implications of smoking. In fact, according to the WHO global report on trends in the prevalence of tobacco use from 2000 to 2025, the greatest progress in reducing rates of smoking is expected to occur in the South East Asia Region, where smoking rates have already declined from an estimated 29% in 2000 to 19% in 2010 and 13% in 2020 (WHO, 2021).

In this review, percentages of parents who believed that THS is harmful ranged from 42.4% to 91%. These data are similar to those emerging from the general population. A Korean survey found that 92.2% of adults held the belief that exposure to THS could have negative effects on children’s health [32]. Winickoff and colleagues (2009) conducted a national random-digit-dial telephone survey to investigate the general population’s beliefs on the health effects of THS. The study showed that 95.4% of individuals who did not smoke, in comparison to 84.1% of smokers, concurred that SHS is detrimental to children’s health. In addition, 65.2% of nonsmokers, as opposed to 43.3% of smokers, acknowledged that THS compromises children’s health [33]. Similarly, Bernhardt and colleagues (2019) [34] conducted a survey to explore the knowledge of the general population about smoking and its negative effects on children’s health in three different contexts: in the same room, in a different room, or in the external space of the house in which the child resides.

The results showed that the respondents underestimated the hazards of THS, and they only linked children’s health issues resulting in respiratory ailments with cigarette smoking in the same surroundings [34]. The same critical issue was underlined by Roberts and colleagues (2016), who surveyed 310 adults in an academic medical center and found that very few respondents perceived risk from THS exposure [35]. Currently, the hazards of THS are not as well documented as those of SHS. This critical issue finds a correspondence in this review, where the overall level of parental knowledge and awareness about THS is lower than that about SHS. In an Iranian study, the authors found that, with regard to SHS, 79.6% of parents fully concurred with its harmful influence on their child’s health, whereas only 42.4% of parents did so with regard to THS [27]. Parents and families have a unique role in protecting children from TSH exposure and ensuring a healthy environment. Few studies demonstrated that increasing parental awareness and knowledge of the impact of THS on their children’s health through different types of educational interventions could positively affect their habits. These behavioral changes translate to trying to quit smoking and reinforcing smoking bans in homes and cars [25,29]. Numerous scientific investigations demonstrated a statistically significant correlation between parents who hold the belief that THS affects their children’s health and the implementation of a prohibition on smoking within their homes [25,27]. Nevertheless, one study highlighted the weak association between knowledge and good practice on THS; this correlation may suggest that parental education alone is not enough to change parents’ behaviors, and further strategies, including smoking cessation programs with pharmacological support, should be considered [17].

Most of the studies collected data from parents and families using a questionnaire developed ad hoc by the authors. This assessment tools’ heterogeneity may complicate the data analysis and comparison. Two studies [18,30] used the BATHS scale validated by Haardörferet et al. (2017) [31], while one paper utilized the parental perceptions of exposure (PPE) tool developed and validated by Myers Gamliel et al. (2018) to shed light on parental smoking habits [29]. The statistical analysis showed good psychometric properties of the PPE tool. Exploratory factor analysis, test–retest reliability, and Cronbach’s alpha coefficient were assessed. Factor analysis produced six elements for PPE, including THS exposure. Myers Gamliel et al. (2018) concluded that this tool may be helpful in understanding parents’ smoking behavior around their children and in explaining discrepancies between parental and objective reports of exposure [36,37]. Dai and colleagues (2021) designed the KAP (knowledge, attitude, and practice) scale based on the WHO KAP guideline, previous KAP scale development studies, the Global Adult Tobacco Survey, and the Health Belief Model. Then, a panel revised the tool, which was tested in a pilot study. The final version had an overall Cronbach’s alpha coefficient of 0.97 [28].

This study presents some limits and strengths. The number of included studies is not high, but this is related to the low number of research papers on the topic. Also, even if several studies enrolled a large number of participants, they adopted a non-random sampling method, and potential selection bias could have occurred since participants were volunteers. Moreover, most of the studies are based on an online survey, and this aspect limits the participation of the population only to those who have access to a smartphone, tablet, or computer. Also, there is a predominance of Asian studies (62.5%), which might raise concerns about different cultural perspectives on the topic; however, the main concepts derived from the Asian studies (parents with higher education are more aware of the harm caused by THS to their children; there is a discrepancy between knowledge and actual good practices; and parental awareness campaigns lead to greater awareness of the health damages caused by THS on children’s health) are also present in the Western studies, supporting the hypothesis that different cultures do not seem to influence this topic significantly. However, further studies are clearly needed, especially for Western countries. For all these aspects, the generalization of the results must be performed cautiously.

On the other hand, to our knowledge, this is the first study aimed at investigating knowledge, beliefs, and behaviors among parents and families about THS. The systematic review by Díez-Izquierdo et al. (2018) considered all aspects of THS, focusing especially on biomarkers of THS, ways of measuring them, and health effects. Moreover, it dates back to 2018, excluding recent important articles [26]. Indeed, the relatively new topic of THS is gaining attention, as shown by the heterogeneity of states where surveys were conducted, which underlines that concern about the negative effects of THS on pediatric health is rising worldwide. Another aspect that deserves attention is that public awareness about the health effects of THS is low and needs to be improved, focusing on actions and recommendations to prevent children’s exposure. This study underlines the importance of conducting more research on this topic and spreading the results.

## 5. Conclusions

To date, there is substantial proof indicating the health dangers that arise from children being exposed to SHS, and there is a growing body of evidence regarding the hazards of THS. As approximately 40% of children globally are regularly subjected to tobacco smoke, primarily due to residing with individuals who smoke, the resultant impact on health is noteworthy [38]. The acute harmful effects of THS on cellular health, both in animal models and in humans, particularly children, are becoming evident. Nonetheless, the long-term consequences on human health still remain an underinvestigated area of research. Moreover, the literature underlined that the understanding of THS and its possible deleterious effects is lacking among the general public [26]. Parents and families have a primary role in protecting children, toddlers, and infants from THS hazards. This review aimed to explore parental knowledge, beliefs, and behaviors on THS, and it showed that percentages of parents who believed that THS is harmful ranged from 42.4% to 91%. It would be useful to adopt a valid and standardized tool in order to gain as much information as possible and especially compare data about parental knowledge, beliefs, and behaviors on THS.

Education and awareness policies need to place more importance on THS. Further research should focus in three main directions: identifying effective educational strategies to improve parents’ knowledge and raise their awareness of the negative effects of THS; analyzing the factors that influence parents to not consider THS harmful in order to change their beliefs; and finally, understanding the resistance and problems faced by parents who are aware of the risks of THS but are unable to improve good practices. Public health institutions and governments should plan interventions based on scientific evidence in order to promote national smoking bans and anti-smoking campaigns. More research aimed to investigate the entity and impact of THS is highly needed.

## Figures and Tables

**Table 1:** Main characteristics of all the included studies.

Authors	Country	Design	Population and Sample Size	Tool	Main Findings	JBI Critical Appraisal Score
Patel et al., 2012 [17]	USA	Pilot study	40 caregivers (18 control–22 intervention group)	Self-report questionnaire	A total of 75% of parents recognized the negative impact of THS * on their child’s health. A brief THS * intervention influenced smokers to change smoking behaviors.	6/13
Drehmer et al., 2014 [25]	USA	Pre–post intervention study	1980 parents	Pre–post survey + computer-aided telephone interview	The belief in the harmful effects of THS * was linked to a tightly enforced ban on smoking in the home and car, as well as efforts to quit smoking. Educating parents could contribute to positive results in controlling tobacco use	6/13
Díez-Izquierdo et al., 2018 [26]	Spain	Cross-sectional	1406 parents with children between 3 and 36 months old	Online survey	A total of 80% of parents held the belief that exposing their children to THS * can be detrimental, and among participants with advanced levels of education, this percentage reached 90%.	5/8
Baheiraei et al., 2018 [27]	Iran	Cross-sectional	1112 families of infants aged 1 year or younger	Questionnaires and face-to-face interviews	Almost half of parents completely agreed with the effects of THS * exposure on their infant’s health.	5/8
Myers Gamliel et al., 2020 [29]	Israel	RCT ***	159 families of children <8 years old randomized into 3 groups	23-item PPE **** online questionnaire	Parental perceptions of exposure were increased significantly post-intervention, indicating that they can be altered.	7/13
Shehab and Ziyab, 2021 [30]	Kuwait	Cross-sectional	536 parents with at least one child aged <18 years	Web-based survey incl. BATHS ***** scale	Parents’ harm and persistence beliefs about THS * were associated with enforcing a strict home smoking ban.	4/8
Xie et al., 2021 [18]	China	Cross-sectional	843 parents and grandparents of children aged 6–13 years	Paper-based survey incl. BATHS ***** scale (Chinese version)	Younger people with higher education levels obtained higher scores.	4/8
Dai et al., 2021 [28]	China	Cross-sectional	145 parents of children with median age of 2.7 (1.2–6.3) years	Standardized questionnaire + KAP ** questionnaire	Discrepancy between knowledge and good practice related to THS *.	6/8

* THS, third hand smoke; ** KAP, knowledge, attitude, and practice; *** RCT, randomized control trial; **** PPE, parental perceptions of exposure; and ***** BATHS, Beliefs About ThirdHand Smoke scale.

## Data Availability

Not applicable.
